# PathVista Integrates Cellular Foundation Models with Multiscale Histopathology for Colorectal Cancer Prognosis and Molecular Stratification

**DOI:** 10.21203/rs.3.rs-11037299/v1

**Published:** 2026-10-08

**Authors:** Usama Sajjad, Abdul Rehman Akbar, Tianyang Wang, Wendy L. Frankel, Metin N. Gurcan, Wei Chen, Muhammad Khalid Khan Niazi

**Affiliations:** 1 Department of Pathology, College of Medicine, The Ohio State University Wexner Medical Center, Columbus, OH, USA; 2 Center for Artificial Intelligence Research, School of Medicine, Wake Forest University, Winston-Salem, NC, USA

## Abstract

Personalized management of colorectal cancer (CRC) requires accurate risk stratification and identification of clinically actionable molecular alterations. However, standard clinicopathologic features often fail to capture the cellular and tumor microenvironmental heterogeneity driving disease behavior. Although routine hematoxylin and eosin (H&E) whole-slide images (WSIs) reflect these patterns, patch-level foundation models may obscure fine-grained cellular morphology and spatial organization. We present **PathVista**, a cell-centered framework that integrates cellular and tissue-level representations for prognostic and molecular stratification. PathVista incorporates **CellSwAV**, a cellular foundation model pretrained on more than 160 million CRC cells and combines spatially aggregated cellular features with broader tissue context. Across **2,568 CRC patients**, PathVista risk scores were significantly associated with **overall survival** (**HR** = **6.65**, **95% CI**: **3.53–12.51**), **disease-free survival** (**HR** = **3.15**, **95% CI**: **1.82–5.45**), and **recurrence-free survival** (**HR** = **3.04**, **95% CI**: **1.76–5.26**), with risk scores remaining independently associated with outcomes after clinicopathologic adjustment. Locked models also generalized to external **TCGA** (**HR** = **5.25**, **95% CI**: **2.29–12.05**) and **SURGEN** (**HR** = **2.89**, **95% CI**: **1.86–4.49**) cohorts. PathVista predicted **microsatellite instability** (**AUC** = **0.91**) and ***BRAF* mutation status** (**AUC** = **0.83**). Across **4,667 patients** spanning CRC, lung, and breast cancers, PathVista demonstrated the potential of cell-centered histopathology for prognostic, molecular, and tumor characterization.

## Introduction

Cells are the fundamental units through which cancer becomes clinically aggressive, as invasion, recurrence, metastasis, immune escape, and therapeutic resistance arise from malignant-cell programs and interactions with immune and stromal compartments in the tumor microenvironment [1–4]. In epithelial cancers, progression can involve epithelial-mesenchymal transition (EMT) activation, proliferative signaling, angiogenesis, invasion, metastasis, and suppression of tumor immunity [1, 4]. Tumor budding is a clinically established example, where isolated tumor cells or small clusters at the invasive front mark an invasive phenotype associated with recurrence, metastasis, and poor survival [2–4]. The tumor microenvironment can also restrain or promote progression: lymphocyte-rich neighborhoods are linked to favorable outcomes, whereas fibrotic, myeloid-rich, and cancer-associated fibroblast–mediated niches can promote immune suppression, T-cell exclusion, treatment resistance, and poor prognosis [1, 5].

Spatial omics has revealed that tumors are organized into conserved multicellular neighborhoods of epithelial, lymphoid, myeloid, stromal, and endothelial cells, with functional programs related to proliferation, hypoxia, antigen presentation, immune-checkpoint activity, prognosis, and immunotherapy response [5–9]. However, these technologies remain costly, complex, and difficult to scale clinically. In contrast, hematoxylin and eosin (H&E) whole-slide images (WSIs) are routinely generated and capture cellular morphology and tissue organization across the entire specimen [5, 6]. Deep learning studies have shown that H&E WSIs contain information relevant to tumor detection, subtyping, molecular alterations, treatment response, recurrence, and survival [10–22]. Current foundation models in computational pathology have transformed slide-level analysis by encoding high-level histomorphological context at the patch-level. However, this coarse-grained abstraction risks masking subtle yet clinically important signals within the tumor microenvironment. Key prognostic drivers, such as early immune cell exclusion, rare cellular niches, and focal subtle nuclear pleomorphism, frequently operate at single cell resolution and can be diluted when features are pooled across an entire patch. Rather than viewing patch-level and cell-level analyses in isolation, they should be considered complementary components of histopathologic analysis. Patch-level representations capture structural tissue topology and tumor stromal architecture, whereas cell-level representations distinguish specific cell states, lineage specific phenotypes, and microscale spatial dynamics. Bridging these two modalities captures the multiscale continuum of the tumor microenvironment, transforming raw WSIs into rich, clinically meaningful representations that reflect both local cellular function and broader tissue architecture.

This cellular view is particularly important in CRC, where MSI-H/dMMR and *BRAF*-mutated tumors exhibit distinct histologic and immune microenvironmental characteristics [23, 24]. MSI-H/dMMR tumors often show right-sided location, mucinous histology, Crohn-like lymphoid reactions, and abundant tumor-infiltrating lymphocytes, while MSI-H/dMMR status predicts benefit from immune-checkpoint blockade [23, 25, 26]. *KRAS* and *BRAF* mutations dysregulate the Ras/Raf/MEK/MAPK axis; *KRAS* mutations predict lack of benefit from anti-*EGFR* therapy, whereas *BRAF V600E* identifies a poor-prognosis subgroup with targeted treatment implications, including combined *BRAF* and *EGFR* inhibition [27–29]. Thus, these clinically actionable alterations may leave detectable cellular and microenvironmental signatures in routine H&E tissue.

To bridge cellular morphology, spatial cellular organization, and broader tissue architecture within a unified WSI framework, we developed **PathVista**, a multiscale computational pathology framework for prognostic and molecular stratification. At its core, PathVista incorporates **CellSwAV**, a cellular foundation model pretrained on more than 160 million individual H&E-derived cells using self-supervised learning [30]. Akin to the systematic approach used by pathologists during tissue examination, PathVista integrates “high-power” CellSwAV-derived cellular representations with “low-power” patch-level tissue features to capture both fine-grained cellular morphology and broader tissue architecture. We evaluate PathVista for CRC overall survival (OS), disease-free survival (DFS), recurrence-free survival (RFS), and MSI, *KRAS*, and *BRAF* mutation prediction. We further assess the transferability of CellSwAV-derived cellular representations to lung and breast cancer tasks and examine associations between PathVista-derived risk scores, attention-associated cellular phenotypes, and tumor-related molecular programs. The overall study design and PathVista framework are summarized in Fig. 1.

## 1. Materials and Methods

### 1.1. Study participant details

This study included H&E-stained WSIs from six clinical cohorts spanning three organ systems: CRC, lung cancer, and breast cancer. The clinical evaluation comprised 4,667 patients, including 2,568 patients with CRC, 1,147 patients with lung cancer, and 952 patients with breast cancer. Separately, 689 human samples from the HEST-1K spatial transcriptomics dataset [31] were used for biological interpretation and were not included in the clinical patient count. The 250 CRC WSIs used for CellSwAV pretraining were obtained from CALGB 89803 (Alliance, detailed below), a multicenter phase III trial involving 38 participating institutions across the United States and providing detailed demographic, clinicopathologic, treatment, recurrence, and survival information [32–34]. These patients were excluded from all downstream model development, cross-validation, and external evaluation. To assess whether PathVista generalizes beyond CRC, we further evaluated the model in lung and breast carcinoma cohorts. All analyses were performed using deidentified retrospective data with available H&E WSIs and corresponding clinical, survival, recurrence, molecular, or subtype labels.

#### 1.1.1. Alliance Colon Cancer OS Cohort

The Alliance cohort was derived from Cancer and Leukemia Group B (CALGB) 89803, a randomized phase III intergroup trial prospectively designed to determine whether adding irinotecan to adjuvant fluorouracil and leucovorin improved survival after complete resection of stage III colon cancer (ClinicalTrials.gov: NCT00003835; CTRP Registry: NCI-2012-01844, Registration Date: April 27, 2004) [32–34]. The trial enrolled 1,264 patients from 38 institutions across the United States and Canada, with participation from CALGB, NCCTG, NCIC CTG, ECOG, SWOG, and the NCI Cancer Trials Support Unit. Patients were followed for approximately eight years, with a median follow up of 7.7 years, providing mature recurrence and survival outcomes. The prospectively curated cohort includes standardized treatment assignments, H&E WSIs, demographic information, detailed clinicopathologic annotations, and longitudinal clinical outcomes, including OS, DFS, and RFS. This geographic, demographic, and clinical breadth supported internal PathVista model development and rigorous evaluation against established prognostic factors.

#### 1.1.2. SURGEN CRC Cohort

The SURGEN CRC cohort comprised 843 patients represented by 1,020 H&E-stained WSIs [35]. For patients with multiple WSIs, one slide was randomly selected to ensure that each patient contributed a single WSI to the analysis. For OS prediction, patients with available survival follow-up were used as an independent external validation cohort, with the locked Alliance-trained models applied without retraining. Model performance was assessed using discrimination metrics, survival risk stratification, hazard ratios (HRs), and concordance index (C-index). Separately, SURGEN cases with available molecular annotations were used for *BRAF* and *KRAS* mutation prediction using within-cohort five-fold cross-validation.

#### 1.1.3. TCGA Colon Adenocarcinoma Cohort (TCGA-COAD)

The TCGA-COADREAD cohort included 461 TCGA-COAD patients and 172 TCGA-READ patients with H&E WSIs and available clinical or molecular data. For OS prediction, the TCGA-COAD cases with available survival information served as an independent external validation cohort, with the locked Alliance-trained models applied without retraining. For molecular prediction, cases from both TCGA-COAD and TCGA-READ with available annotations were included and evaluated using within-cohort five-fold cross-validation. MSI ground-truth labels were obtained from DeepHistology [36], which curated MSI status for TCGA-COADREAD cases. MSI status was formulated as a binary prediction task from routine H&E WSIs.

#### 1.1.4. Non-CRC cohorts for recurrence prediction and tumor subtyping

To assess whether the CRC-pretrained PathVista representation can generalize beyond CRC tissue, we evaluated the model in independent lung and breast cancer cohorts. The invasive lung adenocarcinoma (ILA) cohort included 189 patients with stage I to III ILA who underwent curative intent surgical resection at Wake Forest Baptist Comprehensive Cancer Center between 2008 and 2015, with a minimum follow up of five years [11]. Follow up data included recurrence status and time to recurrence. Within five years, 72 patients (38.1%) experienced disease recurrence. The cohort had a balanced age distribution and included slightly more female patients (56.6%) than male patients (40.7%); sex information was unavailable for five patients (2.6%). The TCGA-NSCLC cohort included 958 patients with H&E WSIs from lung adenocarcinoma and lung squamous cell carcinoma cases and was used for binary lung cancer subtyping (lung adenocarcinoma vs lung squamous cell carcinoma). The TCGA-BRCA cohort included 952 patients with H&E WSIs and was used for breast cancer subtyping (invasive lobular carcinoma vs invasive ductal carcinoma). Separately, for biological interpretation, we used a curated subset of 689 HEST-1K human samples with paired H&E WSIs and spatial transcriptomic profiles to associate CellSwAV clusters with pathway-level gene-expression programs [31]; these samples were not included in the clinical prediction cohorts.

### 1.2. WSI preprocessing and cell extraction

WSIs were preprocessed using a standard computational pathology pipeline consisting of tissue detection, background removal, and feature extraction [37]. For CellSwAV pretraining, individual cells were detected from WSIs using a pretrained cell segmentation model [38]. Each detected cell was represented by its centroid coordinate, and a fixed-size cell-centered image crop was extracted around each centroid. This procedure yielded more than 160,234,871 unlabeled cells for cellular foundation model pretraining.

### 1.3. CellSwAV pretraining

CellSwAV was designed to learn a cellular lexicon from H&E WSIs without manual cell-type annotation. We pretrained a cellular foundation model based on the ResNet-50 architecture using Swapping Assignments between Views (SwAV), an online clustering-based self-supervised learning method. For each cell, two augmented views were generated and passed through the encoder and projection head to produce normalized features. These embeddings were then assigned to a set of learnable prototype vectors (clusters), encouraging cells to cluster together in feature space.

Let $z_{i}^{1}$ and $z_{i}^{2}$ denote the features of two augmented views of cell i, and let C denote the learnable clusters. Cluster prediction probabilities were computed using eq. 1:

(1) $$p_{i}^{\left(v\right)}=\text{softmax}\left(\frac{C^{T}z_{i}^{\left(v\right)}}{\tau }\right),v\in \{1,2\},$$

where τ is the temperature parameter. To avoid collapse of cells into a small number of clusters, balanced cluster assignments were estimated using entropy-regularized optimal transport and solved using the Sinkhorn-Knopp algorithm [39]. The model was optimized using the swapped prediction loss (eq. 2):

(2) $$L_{\text{SwAV}}=-\frac{1}{2B}\sum_{i=1}^{B}\left[q_{i}^{(1)\text{T}}logp_{i}^{\left(2\right)}+q_{i}^{(2)\text{T}}logp_{i}^{\left(1\right)}\right],$$

where $q_{i}^{\left(v\right)}$ is the balanced cluster assignment for view $v,p_{i}^{\left(v\right)}$ is the predicted prototype distribution, and B is the batch size. After pretraining, the projection head was removed, and the cell foundation model was used to extract cell-level features for downstream tasks. The hyperparameters used for CellSwAV pretraining are provided in Supplementary Table 7.

### 1.4. Dual-stream feature extraction and fusion

For each downstream WSI, PathVista constructed a multiscale representation by analyzing the same tissue region at two complementary spatial scales. At the fine cellular scale, the pretrained cell foundation model generated an embedding for each individually detected cell from its cell-centered crop, capturing nuclear and cytoplasmic morphology and local cell-to-cell variation. At the broader tissue-patch scale, the corresponding image patch was encoded using a pretrained pathology foundation model [40] to capture overall tissue architecture. Overall, PathVista represented both microscopic cellular phenotypes and tissue architectural patterns that cannot be fully captured at either scale alone by integrating information from individual cells with the surrounding tissue context.

For each patch j, all cells whose centroids fell inside the patch boundary were grouped into a cell set $CS_{j}$. Cell features within each patch were aggregated using a spatially aware transformer. A learnable classification token was prepended to the sequence, and pairwise cell distances were incorporated as a bias in self-attention (eq. 3):

(3) $$\text{Attention}(Q,K,V)=\text{softmax}\left(\frac{QK^{T}}{\left.\sqrt{d_{k}}\right)}-\lambda D_{j}\right)V,$$

where $D_{j}$ is the pairwise Euclidean-distance matrix calculated from the cell-centroid coordinates within patch $j,d_{k}$ is the dimensionality of the key vectors, and λ controls the contribution of spatial distance. Subtracting the distance term penalizes interactions between spatially distant cells and preferentially emphasizes nearby cellular relationships, while the content-based attention term allows morphologically similar or complementary cells to interact. The output class token $\tilde{c_{J}}$, was then used as the cellular summary for that patch. Although the cell embeddings were summarized using a CLS token, aggregation occurred only after individual cells had been explicitly localized and encoded as separate tokens. In contrast, patch-based models use a fixed spatial grid, causing each patch representation to merge heterogeneous cells, stroma, and background before aggregation and potentially dilute rare or localized cellular signals. The cellular stream therefore preserves cell-specific morphology and spatial relationships, whereas the patch stream captures broader tissue architecture. Combining the two streams integrates biologically localized cellular information with complementary tissue context across spatial scales.

The cellular summary $\tilde{c_{J}}$ was then fused with the tissue-level patch features. Briefly, both feature vectors were projected into a common feature space and combined using bilinear outer-product fusion (eq. 4):

(4) $$u_{j}=\tilde{c_{J}}⊗\tilde{t_{J}},$$

where ${\tilde{c}}_{J}$ is the projected cell features and ${\tilde{t}}_{J}$ is the projected tissue features. The resulting fused feature was projected into a compact patch-level feature and used for slide-level prediction.

### 1.5. Slide-level aggregation and prediction

Fused patch features were aggregated using attention-based multiple instance learning [41]. For a WSI with M fused patch features ${\left\{u_{j}\right\}}_{j=1}^{M}$, the model learned an attention weight for each patch and computed a slide-level representation (eq. 5) as:

(5) $$h_{S}=\sum_{j=1}^{M}a_{j}u_{j},$$

where $a_{j}$ represents the learned task-specific attention weight for patch j. The slide representation was passed to a task-specific prediction head to compute a risk score. Models were trained using weighted binary cross-entropy for the task. For five-year time-dependent binary outcomes, patients who experienced the event within five years were classified as positive, whereas patients who remained event-free with at least five years of follow-up were classified as negative. The hyperparameters used for PathVista evaluation are provided in Supplementary Table 8.

### 1.6. Biological interpretation of PathVista

#### 1.6.1. HEST-based biological annotation of CellSwAV prototypes

To biologically interpret the cellular phenotypes learned by CellSwAV, we used the HEST-1K human dataset, which provides paired H&E WSIs and spatial transcriptomic (ST) profiles of cancer and benign specimens. For each HEST slide, every segmented cell was assigned the expression profile of its nearest ST spot, defined by the Euclidean distance between the cell centroid and the spot centroid in slide coordinates (eq. 6):

(6) $$\text{spot}\left(c\right)=\underset{s\in 𝒮}{\text{argmin}}{\left\|x_{c}-x_{s}\right\|}_{2},\;{𝒞}_{p}=\left\{c:\text{proto}\left(c\right)=p\right\},\;{\bar{e}}_{p,g}=\frac{1}{\left|{𝒞}_{p}\right|}\underset{c\in {𝒞}_{p}}{\sum }e_{\text{spot}\left(c\right),g}.$$

Here, ${\text{x}}_{c}$ and ${\text{x}}_{s}$ denote the slide-coordinate centroids of cell c and ST spot s, respectively; proto (c) is the CellSwAV prototype assigned to cell c; $e_{s,g}$ is the log-normalized expression of gene g at spot s; and ${\overset{‾}{e}}_{p,g}$ is the mean expression of gene g associated with prototype p. Because a single Visium-resolution ST spot (approximately 55μm in diameter) typically contains multiple cells, this procedure constitutes spatial nearest-neighbor label transfer rather than single-cell deconvolution. Thus, all cells whose centroids are closest to the same spot inherit that spot’s expression profile. We explicitly treat this as an approximation imposed by spot-level spatial resolution and as a limitation relative to single-cell transcriptomic measurements.

Expression profiles were aggregated across all cells assigned to each of the C CellSwAV clusters, which constitute the fixed morphological codebook learned during pretraining. This aggregation yielded one mean expression vector, ${\bar{\text{e}}}_{p,}.$, for each prototype. Prototype-level pathway activity was quantified as the mean log-normalized expression of genes belonging to each of 15 gene sets from MSigDB [42]. For each pathway, activity scores were z-standardized across all clusters. Enrichment was then evaluated relative to the remaining clusters using a Wilcoxon rank-sum test, with Benjamini-Hochberg correction applied across the 15 pathways (Supplementary Table 9) for each prototype. Each prototype was assigned a dominant pathway identity from its standardized enrichment profile. The resulting prototype-to-pathway mapping was computed once using HEST and retained as a fixed lookup table for all downstream H&E cohorts. Consequently, downstream prototype and pathway assignment required only H&E images and did not require paired transcriptomic data or cohort-specific re-annotation.

For attention-based biological interpretation, patches within each WSI were ranked according to their learned PathVista attention weights, and the top 10% highest-attention patches were selected. Cells within these regions were assigned to their corresponding CellSwAV prototypes using the fixed HEST-derived prototype-to-pathway mapping. Prototype representation within high-attention regions was compared between PathVista-predicted high-risk and low-risk groups using two-sided Mann–Whitney U tests. P values were adjusted across prototypes using the Benjamini–Hochberg procedure, with FDR q < 0.05 considered statistically significant.

#### 1.6.2. TCGA-based pathway analysis of PathVista risk groups

Separately, pathway scores from the TCGA-COAD cohort were used to determine whether pathway-level biology was preserved within PathVista-predicted risk groups. Actual high-risk patients were those who died within five years, whereas actual low-risk patients were those who remained alive with at least five years of follow-up. Predicted risk groups were defined using the prespecified cutoff established in the Alliance development cohort. For each pathway, patient-level pathway scores were aligned between the actual and predicted risk assignments. For the high-risk analysis, the observed pathway score was retained for patients correctly predicted as high risk; for incorrectly classified patients, the pathway score was replaced with the mean pathway score across all patients predicted as high risk. An analogous procedure was applied to the low-risk analysis using the mean pathway score across all patients predicted as low risk. Spearman correlation was then calculated between the actual and prediction-aligned pathway-score vectors. P-values were adjusted for multiple comparisons using the Benjamini–Hochberg procedure, and FDR-adjusted p values below 0.05 were considered statistically significant.

### 1.7. Baseline models and experimental design

PathVista was compared with established deep learning methods, including ABMIL [41], TransMIL [43], CLAM-SB [44], CLAM-MB [44], RRT-MIL [45], FR-MIL [46], and MHIM-MIL [47]. All baseline methods used the same tissue-level foundation-model features to ensure that performance differences reflected the added contribution of cell foundation model cellular features. Internal experiments were evaluated using five-fold cross-validation, and external OS evaluation was performed on independent cohorts where available. For the Alliance development cohort, Cox proportional hazards and Kaplan–Meier analyses were performed using out-of-fold PathVista probabilities, such that each patient’s probability was generated by a model that had not been trained on that patient. For the external TCGA-COAD and SURGEN cohorts, probabilities were generated using the locked models developed on the Alliance cohort, without retraining or threshold adjustment on the external datasets. Models and decision thresholds were established using the Alliance development cohort and were applied to the independent TCGA and SURGEN cohorts. All dataset partitioning was performed at the patient level, and all WSIs from the same patient were assigned to the same partition. For patients with multiple WSIs, one slide was randomly selected to ensure that each patient contributed a single WSI to the analysis. No patient or WSI used for PathVista pretraining was included in any downstream validation or test set.

### 1.8. Statistical analysis

Classification performance was evaluated using area under the receiver operating characteristic curve, balanced accuracy (accuracy), sensitivity, and specificity. All internal survival analyses used out-of-fold predicted probabilities. Survival performance was evaluated using Kaplan-Meier analysis, log-rank testing, Cox proportional hazards modeling, hazard ratios with 95% confidence intervals, and concordance index. Five-year binary classification metrics were calculated only among patients whose five-year outcome status was ascertainable. For cross-validation experiments, performance was reported as mean ± standard deviation across folds. For external cohorts, confidence intervals were estimated using bootstrap resampling. For each endpoint, a multivariable Cox proportional hazards model jointly included the PathVista-derived risk score and the prespecified clinicopathologic variables listed in Section 2.2. For all internal and external survival analyses, Cox proportional hazards models used the continuous PathVista-predicted probability without dichotomization. Hazard ratios therefore represent the change in hazard associated with a one-unit increase in the predicted probability. Dichotomization at the prespecified threshold of 0.5 was used only for Kaplan–Meier visualization and log-rank analysis. Hazard ratios, 95% confidence intervals, and p values were estimated for each variable while adjusting for all other variables included in the model. Pathway and attention analyses were performed to evaluate whether PathVista-derived risk scores were associated with biologically coherent cellular and microenvironmental programs.

## 2. Results

### 2.1. PathVista improves CRC OS prediction and generalizes across external cohorts

We benchmarked PathVista against seven established deep learning baseline methods trained using histopathology foundation-model features, including ABMIL [41], TransMIL [43], CLAM-SB [44], CLAM-MB [44], RRT-MIL [45], FR-MIL [46], and MHIM-MIL [47], for CRC OS prediction across one internal cohort and two independent external cohorts. Across the combined evaluation, PathVista achieved the highest mean accuracy, and mean C-index, demonstrating improved discrimination, case-level classification, and prognostic risk ranking compared with existing patch-based deep learning methods (Fig. 2A–C).

On the internal Alliance cohort, PathVista achieved the best overall performance for five-year OS prediction, with an AUC of **0.70** ± **0.06** and accuracy of **66.80%** (Fig. 2D,G). This exceeded the strongest baseline accuracy, CLAM-MB at **63.28%**, and the strongest baseline AUC, ABMIL at **0.67** (Supplementary Table 1). These results suggest that the cell-level representation learned by PathVista improves both discrimination and clinically meaningful case-level classification within the training-domain cohort.

Additionally, PathVista retained strong performance on independent external datasets, supporting cross-cohort generalizability. On the external SURGEN cohort, PathVista achieved an accuracy of methods (**65.87%**, 95% CI: 60.30 –71.80%) and the highest C-index (**0.6558**, 95% CI: 0.5995–0.7074), while maintaining competitive AUC (0.6780, 95% CI: 0.611 – 0.743) (Fig. 2F,I). Although ABMIL produced the highest AUC, its performance was highly imbalanced, with very high sensitivity (**96.84%**) but extremely low specificity (**8.33%**), limiting its practical utility for risk stratification (Supplementary Tables 2, 3). In contrast, PathVista maintained a more balanced sensitivity–specificity and produced a significant survival separation with HR **2.8916** (95% CI: 1.8632 – 4.4876). PathVista also generalized to the external TCGA-COAD cohort, where it achieved the highest accuracy (**66.15%**, 95% CI: 57.85 – 74.14%) and the highest C-index (**0.6088**, 95% CI: 0.5523 – 0.6627) among all compared methods (Fig. 2E,H). Its AUC (**0.6760**, 95% CI: 0.5723 – 0.7704) was comparable to that of the strongest baseline, ABMIL (**0.6449**), while PathVista provided stronger prognostic stratification, with HR **5.2525** (95% CI: 2.2891–12.0523) and outperforms ABMIL by 6% in accuracy. These external validation results are particularly important because these cohorts differ from the Alliance cohort in data source, tissue processing, and case composition, yet PathVista maintained the best patient-level risk ranking and case-level accuracy. Although absolute discrimination in the external cohorts was moderate, PathVista provided the strongest overall performance across prognostic ranking, balanced case-level classification, and survival stratification relative to the evaluated baseline methods. Because all models used identical UNI-v2 tissue embeddings, the ABMIL comparison provides the most direct assessment of the additional cellular branch.

We next evaluated whether PathVista-derived risk scores could stratify patients into clinically meaningful high-risk and low-risk groups using Kaplan–Meier analysis. In the internal Alliance cohort, PathVista produced clear and statistically significant separation between predicted risk groups across multiple survival endpoints. Patients assigned to the high-risk group had worse OS than those assigned to the low-risk group, with strong separation of the survival curves over follow-up time ($p=1.441\times 10^{-10}$; Fig. 3i). Similar separation was observed for DFS ($p=1.139\times 10^{-6}$; Fig. 3ii) and RFS ($p=1.648\times 10^{-6}$; Fig. 3iii), indicating that PathVista captures prognostic morphology associated not only with mortality but also with disease progression and recurrence.

Importantly, this risk stratification was preserved in independent external cohorts. In the TCGA-COAD cohort, PathVista significantly separated high-risk and low-risk patients for OS despite differences in cohort source and tissue preparation ($p=6.569\times 10^{-5}$; Fig. 3iv). The model also generalized to the SURGEN external cohort, where predicted high-risk patients again showed significantly worse survival than predicted low-risk patients ($p=1.031\times 10^{-3}$; Fig. 3v). These external validation results support the robustness of PathVista-derived risk scores across independent CRC datasets.

Together with the C-index analysis (Fig. 2C), the Kaplan–Meier results show that PathVista-derived risk scores provide clinically meaningful patient stratification across internal and external cohorts, supporting the generalizability of cell-level representation learning for CRC prognosis.

### 2.2. PathVista risk scores remain independently associated with CRC outcomes after clinicopathologic adjustment

Tumor behavior is shaped by cellular and microenvironmental features that may not be fully represented by individual clinicopathologic variables. We therefore evaluated whether the PathVista-derived risk score provides prognostic information relative to established factors, including tumor invasion, mucinous histology, vascular invasion, extramural venous invasion, perineural invasion, lymphocytic response, treatment arm, age, sex, performance status, positive lymph nodes, and tumor differentiation across OS, DFS, and RFS.

Across all three endpoints, the PathVista risk score remained independently associated with patient outcome after adjustment for the evaluated clinicopathologic variables in the multivariable Cox models (Fig. 4). For OS, the PathVista risk score was associated with a mortality risk (HR = 6.647, 95% CI: 3.531–12.511, *p* < 0.001). The association remained consistent for DFS (HR = 3.147, 95% CI: 1.817–5.449, *p* < 0.001) and RFS (HR = 3.043, 95% CI: 1.762–5.255, *p* < 0.001).

Several individual clinicopathologic covariates were not statistically significant in the fitted multivariable models, whereas the PathVista risk score retained an independent association with outcome across OS, DFS, and RFS. Tumor invasion and positive lymph nodes were not significantly associated with these endpoints in the present models. Perineural invasion showed significant associations across all three outcomes, while lymphocytic response was associated with OS and DFS but not RFS. Overall, these multivariable analyses indicate that the PathVista risk score provides prognostic information that is independent of the evaluated clinicopathologic variables, including tumor- and node-related features.

### 2.3. PathVista risk scores preserve pathway structure within predicted risk groups and reveal exploratory attention-associated cellular patterns

Separately from the HEST-based prototype annotation analysis, we used pathway scores from the TCGA-COAD cohort to assess whether the OS risk scores learned by PathVista preserved pathway-level biology within predicted risk groups. This analysis was performed separately from the HEST-1K spatial transcriptomic analysis used to identify pathways enriched in PathVista cellular clusters. Specifically, we correlated pathway scores between actual high-risk and predicted high-risk patients and, separately, between actual low-risk and predicted low-risk patients using Spearman correlation analysis. In the high-risk group, PathVista-derived OS risk preserved strong positive correlations (Fig. 5, left). The strongest correlations were observed for **TGF**-𝛃 **response** $\left(\rho =0.569,\text{FDR}=2.3\times 10^{-12}\right)$, **IFN**-𝛄 **response** $\left(\rho =0.570,\text{FDR}=2.3\times 10^{-12}\right)$, and **activated dendritic cells** $\left(\rho =0.542,\text{FDR}=3.0\times 10^{-11}\right)$, with additional significant correlations for **macrophage regulation, wound healing, proliferation, homologous recombination defects, RNA-based stemness, CD8 T cells**, and **Wnt**/𝛃-**catenin signaling** (all FDR < 0.05).

A similar pattern was observed for the low-risk group (Fig. 5, right), again showing that PathVista preserved pathway structure within correctly predicted patients. The strongest correlations in this group included **activated dendritic cells** $\left(\rho =0.591,\text{FDR}=3.1\times 10^{-13}\right)$, **TGF**-𝛃 **response** $\left(\rho =0.524,\text{FDR}=2.6\times 10^{-10}\right)$, **IFN-𝛄 response** $\left(\rho =0.454,\text{FDR}=9.0\times 10^{-8}\right)$, with significant correlations also observed for macrophage regulation, wound healing, proliferation, homologous recombination defects, RNA-based stemness, CD8 T cells, and **Wnt**/𝛃-**catenin signaling** (all FDR < 0.05). Together, these results indicate that PathVista-derived OS risk scores do not merely separate patients statistically but retain pathway-level structure within both predicted high-risk and predicted low-risk groups.

We then localized the morphologic basis of this signal by examining **high-attention regions** and the cells contained within them. Exploratory analysis of high-attention regions identified nominal differences in prototypes mapped to proliferation, angiogenesis, hypoxia, and MYC-target programs in predicted high-risk cases, and to immune-response and TGF-β programs in predicted low-risk cases. However, individual prototype-level differences did not remain significant after correction for multiple comparisons (FDR q < 0.05), and these findings should therefore be interpreted as exploratory.

These findings are biologically plausible in the context of CRC. Proliferation and angiogenesis are established cancer hallmark programs, while hypoxia promotes dysfunctional vascularization, EMT-like behavior, and metastasis [1, 48]. MYC activation is also linked to cancer growth, microenvironmental remodeling, and immune evasion [49]. In contrast, dense immune infiltration, particularly CD3+, CD8+ TILs are associated with improved clinical outcome in CRC [50, 51]. TGF-β signaling is context dependent, with epithelial tumor-suppressive roles in early disease and stromal, immune-regulatory, and pro-progression roles in advanced or microenvironmental contexts [52–54]. Together, the FDR-corrected TCGA pathway analysis supports associations between PathVista-derived risk scores and molecular and microenvironmental programs, while the attention-based prototype analysis provides exploratory spatial context for these associations.

### 2.4. PathVista predicts MSI status and *KRAS/BRAF* mutations from routine CRC H&E images

MSI status and *KRAS/BRAF* mutations are clinically important biomarkers in CRC because they guide immune checkpoint blockade eligibility, anti-EGFR treatment decisions, and targeted therapy strategies. We therefore evaluated whether PathVista could predict these molecular alterations directly from routine H&E WSIs and compared its performance with established deep learning models trained using foundation-model features. For **MSI prediction**, PathVista achieved the highest AUC (**91.38 ± 3.23%**), high balanced accuracy (**80.70** ± **4.97%**) and sensitivity (**76.06** ± **12.79%**) among the evaluated methods (Fig. 6A, 6D, Supplementary Table 4). This performance is clinically relevant because MSI-H/dMMR status identifies patients who may benefit from immune checkpoint blockade. For ***BRAF* mutation prediction**, PathVista achieved the highest balanced accuracy (**77.83** ± **7.12%**) and sensitivity (**77.58** ± **9.51%**) (Fig. 6B, 6E, Supplementary Table 5). Although CLAM-SB and CLAM-MB showed slightly higher AUC values, PathVista provided a more clinically useful sensitivity–specificity balance for identifying *BRAF*-mutant cases. This is important because *BRAF* V600E defines a poor-prognosis molecular subgroup with targeted treatment implications. For ***KRAS* mutation prediction**, performance was modest across all methods, suggesting that *KRAS*-associated morphology is less separable from H&E than MSI or BRAF. Nevertheless, PathVista achieved an AUC of 60.69% and highest balanced accuracy (**59.10** ± **3.77%**) (Fig. 6C,6F, Supplementary Table 6), reflecting a more balanced prediction profile than competing methods.

### 2.5. CRC-pretrained CellSwAV representations transfer to lung and breast cancer tasks

To evaluate whether cellular representations learned from CRC H&E images generalize beyond the pretraining organ, we applied PathVista to three independent non-CRC tasks: lung cancer five-year recurrence prediction, TCGA-NSCLC subtyping, and TCGA-BRCA subtyping. For lung cancer recurrence prediction, PathVista achieved the highest AUC and balanced accuracy among the evaluated methods (Fig. 7A, 7D). This result supports the transferability of CRC-pretrained cellular representations to a clinically relevant outcome task in an independent organ system, suggesting that PathVista captures recurrence-associated cellular and microenvironmental patterns that are not restricted to CRC. We next evaluated PathVista on tumor subtyping tasks in TCGA-NSCLC and TCGA-BRCA. In both cohorts, PathVista performed within the range of established deep learning methods (Fig. 7B, 7C, 7E, 7F). These findings indicate that the cellular representation learned from CRC tissue can be applied to distinct tumor types and organ systems on morphology-driven classification tasks. Overall, PathVista retained performance across lung and breast cancer cohorts despite being pretrained on CRC H&E images. These results support the cross-organ transferability of PathVista-derived cell-level representations for both recurrence prediction and tumor subtyping from routine H&E WSIs.

## 3. Discussion

Spatial omics have established that tumors are organized cellular ecosystems in which malignant, immune, stromal, and endothelial populations form recurrent neighborhoods with prognostic and therapeutic relevance. However, routine clinical deployment of these technologies remains limited by cost, technical complexity, and scalability. Motivated by these observations, we developed PathVista, that integrates a novel cell foundation model (CellSwAV) designed to determine whether routine H&E WSIs retain cellular and microenvironmental signals that reflect biologically meaningful tumor organization. CellSwAV learns a cellular lexicon directly from individual H&E-derived cells and PathVista integrates these features with tissue architecture, providing a scalable framework for outcome, molecular, and cross-organ prediction from standard histology.

The prognostic results support the value of modeling H&E at cellular resolution. In CRC, PathVista stratified patients across OS, DFS, and RFS in the Alliance cohort; the PathVista-derived risk score was also strongly associated with OS, DFS, and RFS, with HR = 6.647, HR = 3.147, and HR = 3.043, respectively and retained prognostic performance in two independent external cohorts, TCGA and SURGEN. This external validation is important because these cohorts differ in data source, tissue processing, and case composition, yet PathVista maintained patient-level risk ranking and survival stratification across settings. In SURGEN, PathVista achieved the highest C-index among compared methods and separated high- and low-risk patients with HR = 2.8916. In TCGA cohort, PathVista again achieved the highest C-index and showed stronger prognostic stratification, with HR = 5.2525. These external results suggest that the learned cellular risk signal is not restricted to the training-domain cohort, but reflects reproducible morphologic features associated with aggressive CRC biology. These associations were stronger and more consistent than most individual clinicopathologic variables evaluated in the same cohort, including tumor invasion and positive lymph nodes. Together, the internal and external results suggest that PathVista captures a broader morphologic phenotype of tumor aggressiveness that is not fully represented by individual TN-related features or standard pathology descriptors.

The biological analyses provide further support for this interpretation. Exploratory attention-based analysis identified nominal differences in cellular prototypes mapped to proliferation, angiogenesis, hypoxia, and MYC-target programs in high-risk regions and immune-response and TGF-β programs in low-risk regions. These prototype-level differences did not remain significant after multiple-testing correction and therefore require validation in independent cohorts. Although these nominal patterns are biologically plausible, they require validation in independent cohorts. Proliferation, angiogenesis, hypoxia, and MYC signaling have established roles in aggressive tumor behavior, whereas immune-rich microenvironments are generally associated with more favorable outcomes in CRC. The TGF-β association should be interpreted in a context-dependent manner, given its epithelial growth-suppressive roles in some settings and its stromal, immune-regulatory, and pro-progression roles in others. Overall, the FDR-corrected TCGA pathway analysis supports associations between PathVista-derived risk scores and molecular and microenvironmental programs, whereas the attention-based prototype analysis provides exploratory spatial context for these associations.

The molecular prediction results further suggest that clinically relevant genomic alterations are associated with detectable morphologic and microenvironmental signatures in both the tumor and its microenvironment on H&E-stained tissue sections. PathVista achieved high discrimination for MSI prediction, with an AUC of 91.38%, consistent with the known immune-rich and morphologically distinctive features of MSI-H/dMMR CRC. This finding is clinically relevant because MSI-H/dMMR status identifies patients who may benefit from immune checkpoint blockade. PathVista also improved balanced classification for *BRAF* mutation, a poor-prognosis molecular subgroup with targeted treatment implications. In contrast, *KRAS* prediction was more modest across methods, suggesting that *KRAS*-associated morphology may be less visually separable on routine H&E or more heterogeneous across tumor contexts. This distinction is biologically plausible, as not all molecular alterations are expected to produce equally detectable cellular phenotypes.

The cross-organ experiments suggest that the learned cellular lexicon is not restricted to CRC. Although PathVista was pretrained on CRC tissue, it retained predictive utility for lung cancer recurrence prediction and performed within the range of established methods for NSCLC and BRCA subtyping. This suggests that some prognostically relevant cellular programs, including proliferation, immune infiltration, hypoxia, and stromal remodeling, and morphologic features of tumor aggressiveness such as necrosis and mitoses, are shared across epithelial malignancies. At the same time, organ-specific architecture remains important, supporting the integration of cellular representations with tissue-level context rather than modeling cells in isolation.

This study has limitations. All analyses were retrospective and depended on available clinical, survival, recurrence, molecular, and subtype labels. H&E-based mutation prediction should not be interpreted as a replacement for molecular testing, but ratser as a potential screening or morphologic-association tool. In addition, pathway and attention analyses support biological plausibility but remain correlative and do not establish causal mechanisms. Future studies should validate PathVista prospectively, test the model across broader multi-institutional cohorts, and directly compare learned cellular phenotypes with spatial omics, or multiplex immunofluorescence data to determine whether PathVista prototypes correspond to reproducible malignant, stromal, and immune cell states.

In summary, PathVista demonstrates that unsupervised cellular representation learning from routine H&E WSIs can capture biologically meaningful signals associated with prognosis, molecular alterations, and cross-organ tumor classification. These findings support a cell-centered approach to computational pathology in which slide-level predictions are linked to cellular morphology, tissue organization, and pathway-level tumor biology.

## Supplementary Material

Supplementary Files

This is a list of supplementary files associated with this preprint. Click to download.

- supplementarymaterial.docx

## Figures and Tables

**Figure 1 |:**
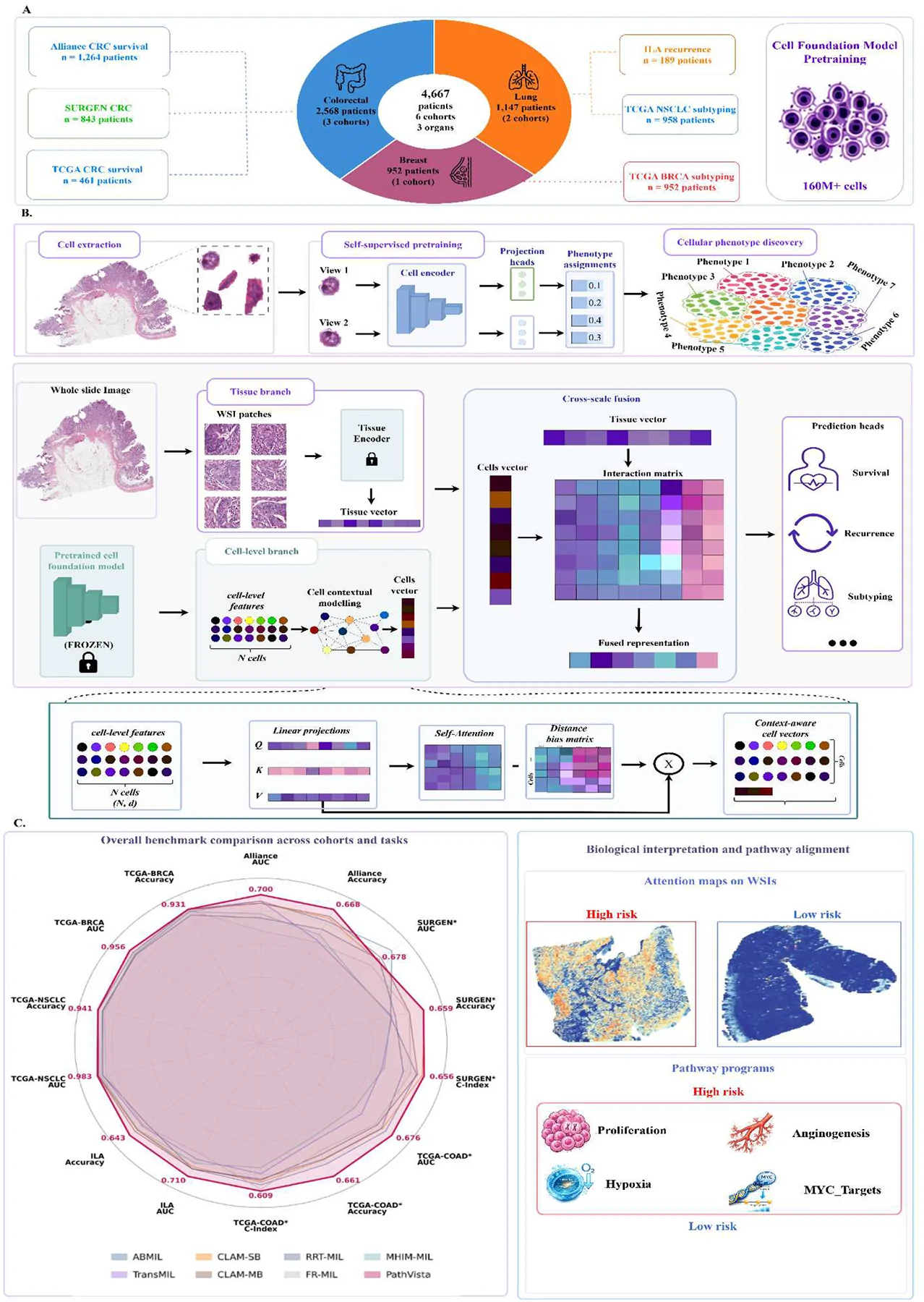
Overview of the PathVista framework, study design, and multi-organ cohort composition. Schematic overview of **PathVista**. **A**, Cohort composition across 4,667 patients from six cohorts and three organs, including CRC OS cohorts from Alliance, SURGEN, and TCGA; invasive lung adenocarcinoma (ILA) recurrence; Non-Small Cell Lung Cancer (NSCLC) subtyping; and Breast Cancer (BRCA) subtyping. **B**, CellSwAV pretraining and downstream slide-level prediction workflow, in which cells are extracted from H&E WSIs and used to train a cell foundation model through self-supervised learning without manual cell-type annotations. Then, the pretrained cell foundation model generates cellular features and cell maps, which are combined with tissue-context features for clinical tasks including OS, RFS, DFS, genetic mutation prediction, and tumor subtyping. **C**, Overall benchmark comparison of PathVista against established deep learning and foundation-model baselines across internal and external validation cohorts.

**Figure 2 |:**
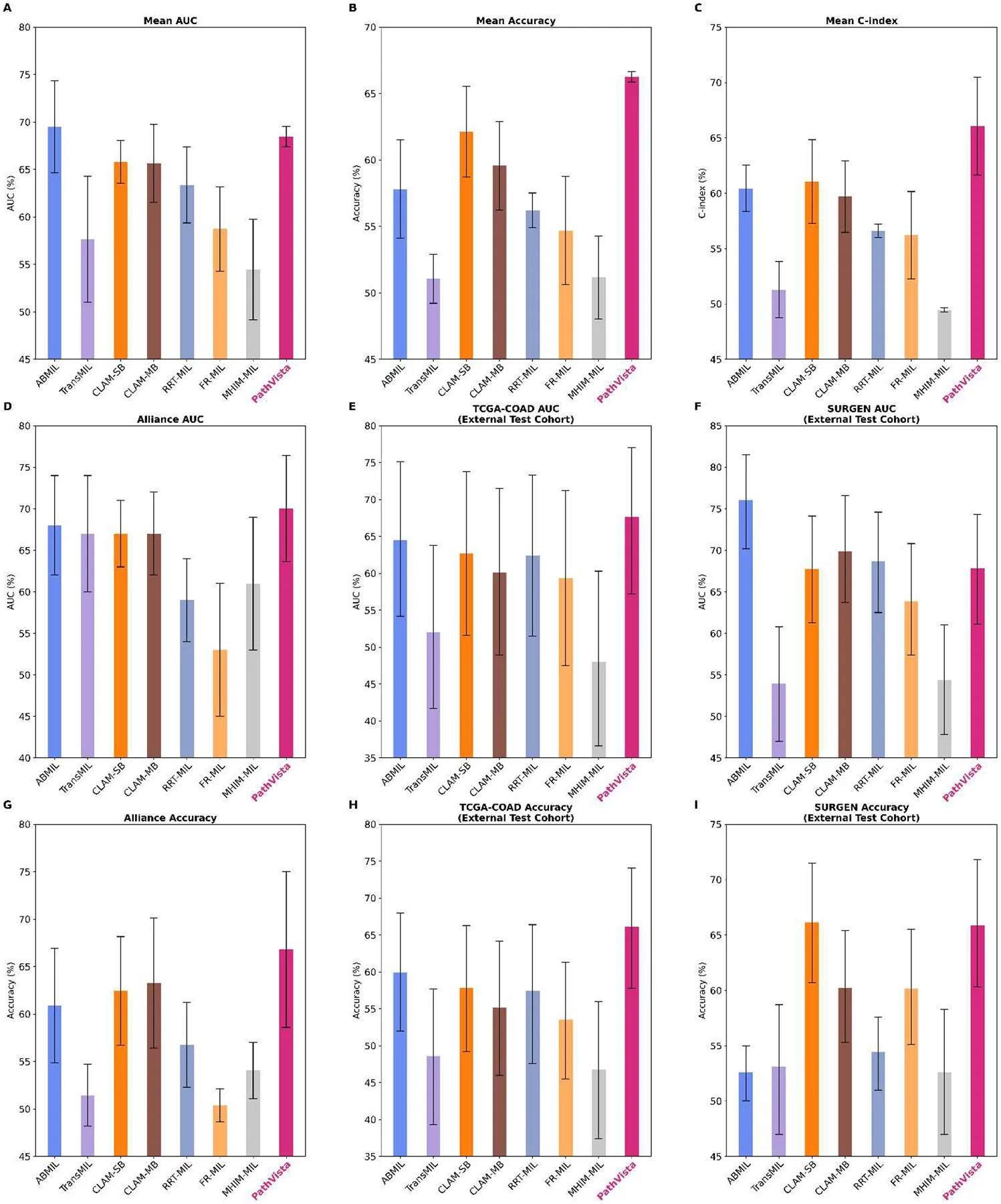
Benchmark comparison of PathVista against established WSI-based deep learning methods for CRC OS prediction. Performance of PathVista was compared with ABMIL, TransMIL, CLAM-SB, CLAM-MB, RRT-MIL, FR-MIL, and MHIM-MIL across internal and external CRC cohorts. **A-C**, Mean performance across cohorts for AUC, balanced accuracy (accuracy), and C-index. Across cohorts, PathVista achieved the highest overall mean accuracy, C-index indicating improved classification performance, and prognostic ranking compared with baseline methods. **D-F**, Cohort-specific AUC on the internal Alliance cohort and external TCGA, SURGEN cohorts. **G-I**, Cohort-specific accuracy on the internal Alliance cohort and external TCGA, SURGEN cohorts.

**Figure 3 |:**
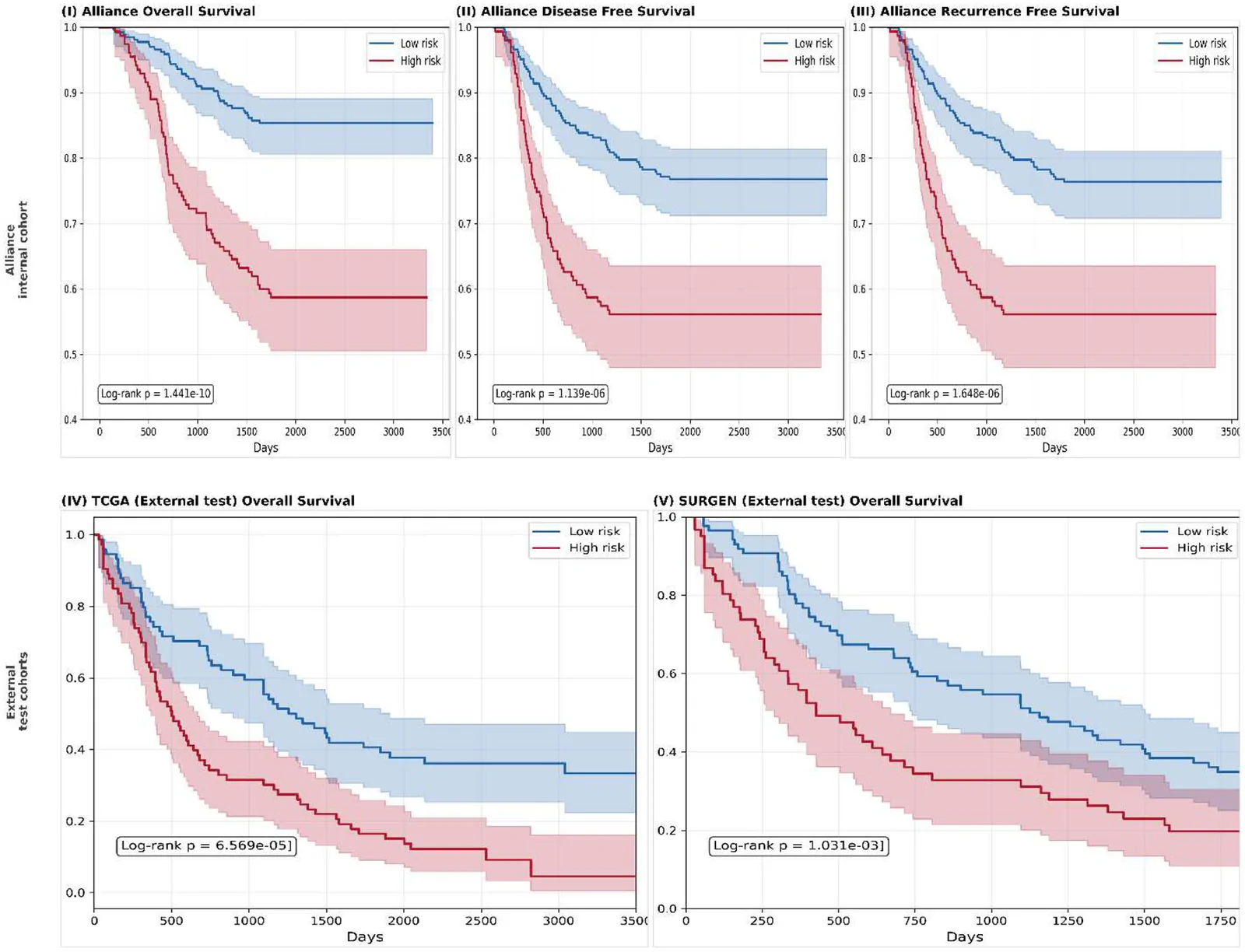
Kaplan–Meier survival stratification across internal and external cohorts. Kaplan–Meier analysis of PathVista-predicted high-risk and low-risk groups across different endpoints. In the internal Alliance cohort, PathVista significantly stratified patients for **OS** $\left(p=1.441\times 10^{-10}\right)$, **DFS** $\left(p=1.139\times 10^{-6}\right)$, and **RFS** $\left(p=1.648\times 10^{-6}\right)$. External validation showed significant separation between predicted high-risk and low-risk patients for **OS** in TCGA cohort ($p=6.569\times 10^{-5}$) and SURGEN ($p=1.031\times 10^{-3}$). Shaded regions indicate confidence intervals around the Kaplan–Meier estimates. Risk groups were defined using PathVista-derived case-level risk scores.

**Figure 4 |:**
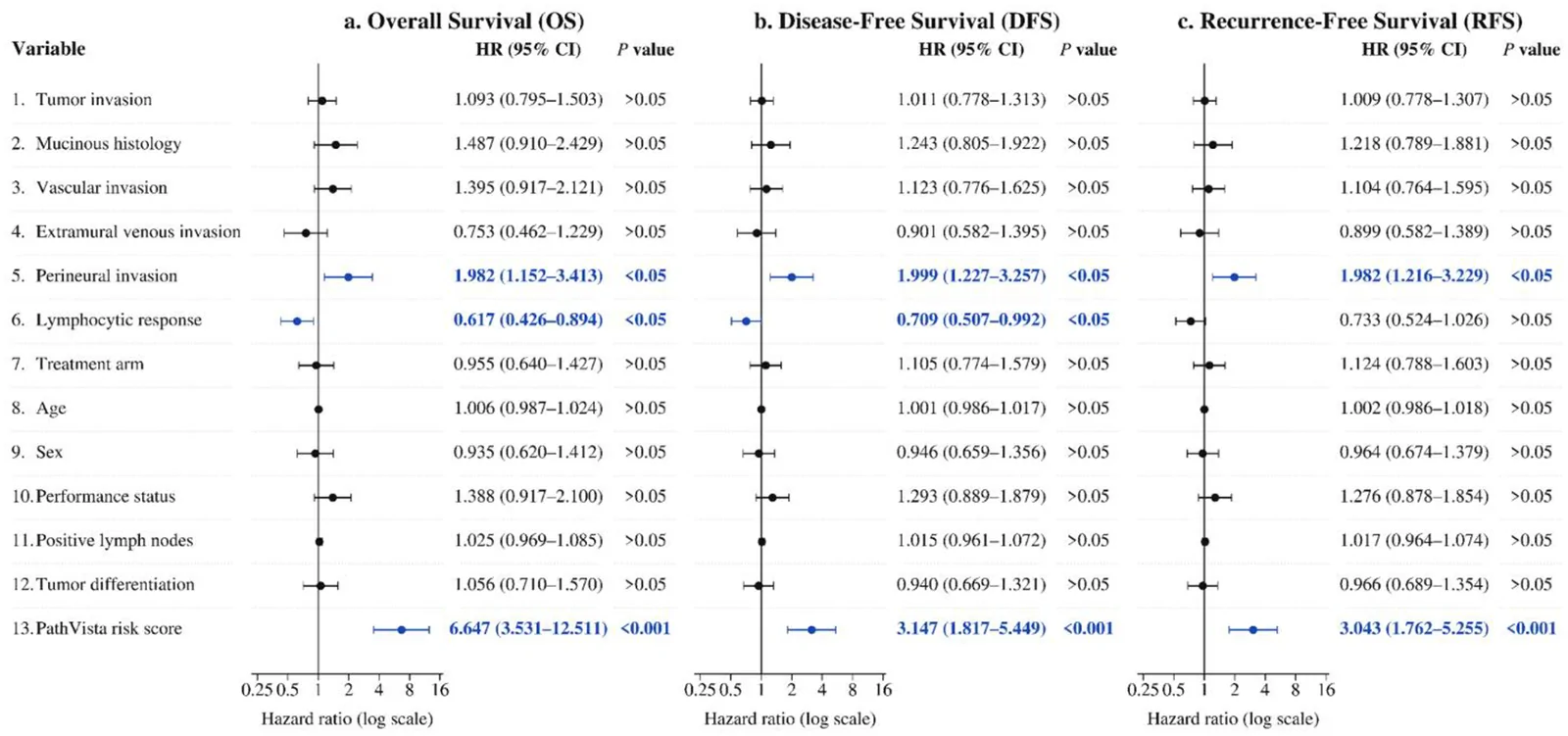
PathVista risk score provides independent prognostic information beyond evaluated clinicopathologic variables across OS, DFS, and RFS. The PathVista risk score represents the continuous predicted probability of event within five years and was entered into the Cox models without dichotomization. Forest plots showing hazard ratios (HRs) and 95% confidence intervals from multivariable Cox proportional hazards models jointly including the PathVista risk score and the evaluated clinicopathologic variables for **a, OS; b, DFS**; and **c, RFS**. The vertical reference line indicates HR = 1, and HRs are shown on a log scale. Across all three endpoints, these findings suggest that PathVista captures prognostic morphologic information that is not fully represented by the evaluated clinicopathologic variables: **OS HR** = **6.647** (**95% CI: 3.531–12.511, *p*** < **0.001**), **DFS HR** = **3.147** (**95% CI: 1.817–5.449, *p* < 0.001)**, and **RFS HR = 3.043 (95% CI: 1.762–5.255, *p* < 0.001)**. Several individual clinicopathologic covariates were not statistically significant in the fitted multivariable models, whereas the PathVista risk score remained independently associated with all three survival endpoints. Perineural invasion was consistently associated with worse outcomes, while lymphocytic response showed a protective association for OS and DFS.

**Figure 5 |:**
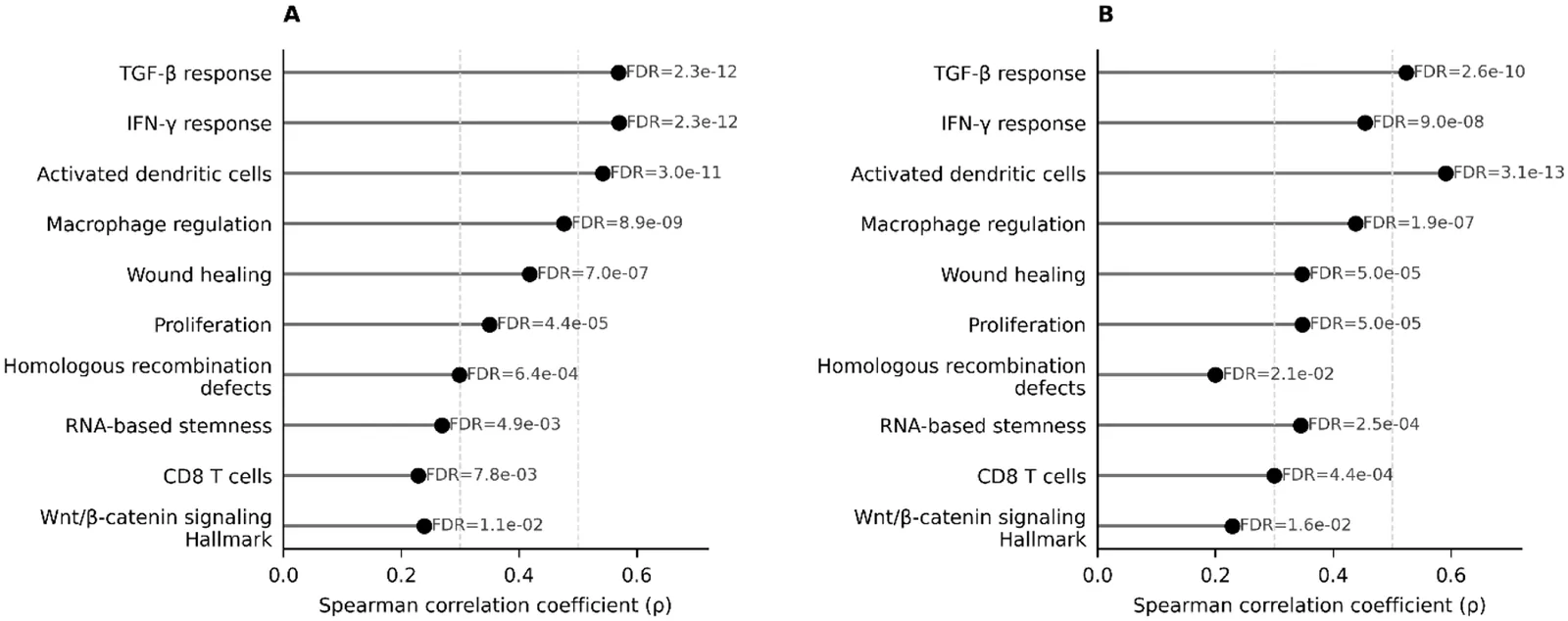
PathVista risk groups preserve pathway-level biology. Spearman correlation between pathway activity profiles in **actual** and **PathVista-predicted** risk groups for OS in TCGA cohort. The left panel shows correlations for the **high-risk** group, and the right panel shows correlations for the **low-risk** group. In both analyses, positive correlations were observed across multiple pathways, with significance assessed using **FDR-adjusted p values**. The pathway signatures used for this TCGA-COAD analysis are listed in Supplementary Table 10.

**Figure 6 |:**
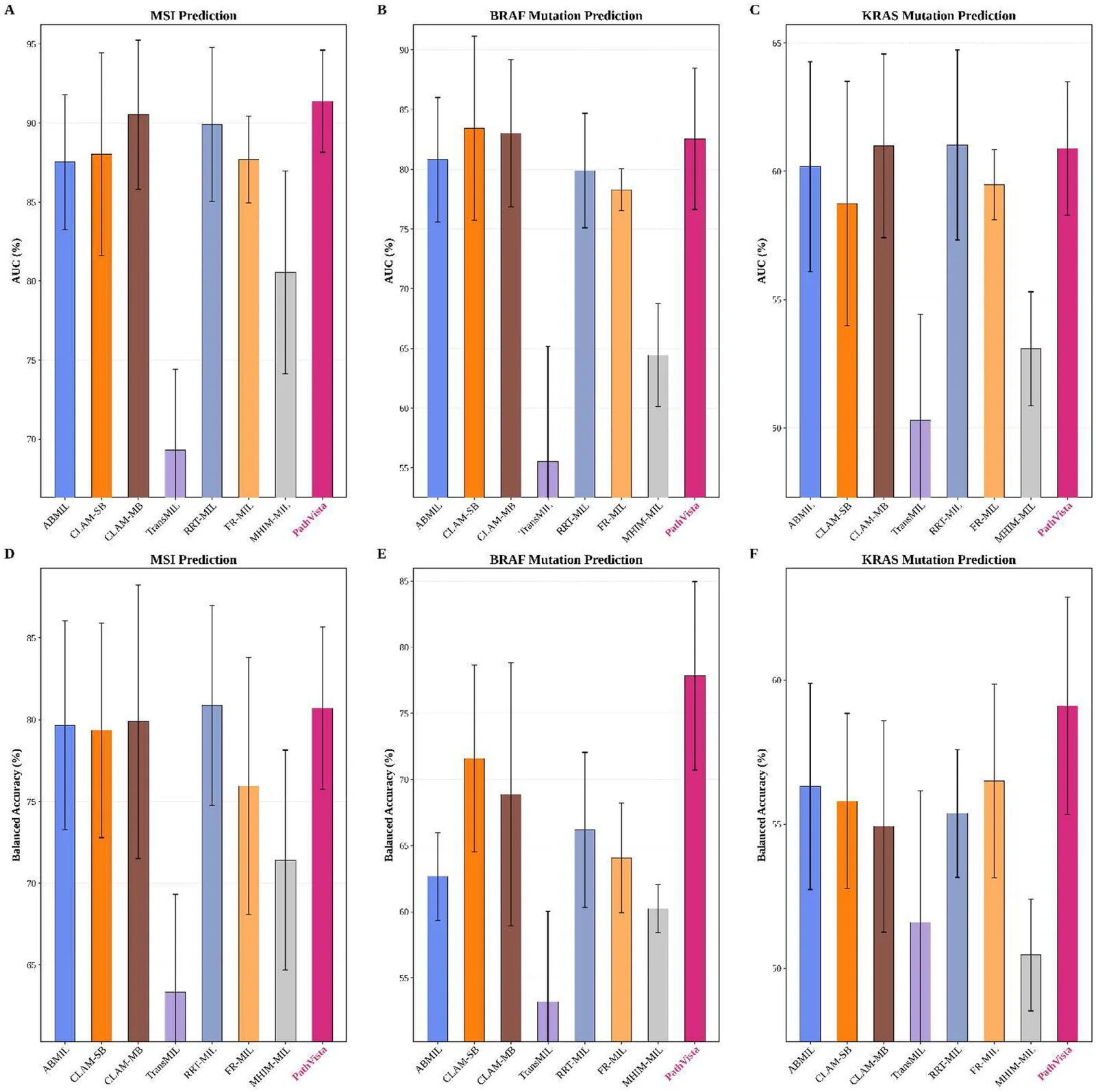
Benchmark comparison of PathVista against established WSI-based deep learning methods for clinically actionable MSI, *BRAF*, and *KRAS* alterations. **A-C**, Mean AUC across five-fold cross-validation for MSI, *BRAF*, and *KRAS* prediction, respectively. **D-F**, Balanced accuracy for the same prediction tasks. Error bars indicate standard deviation across folds.

**Figure 7 |:**
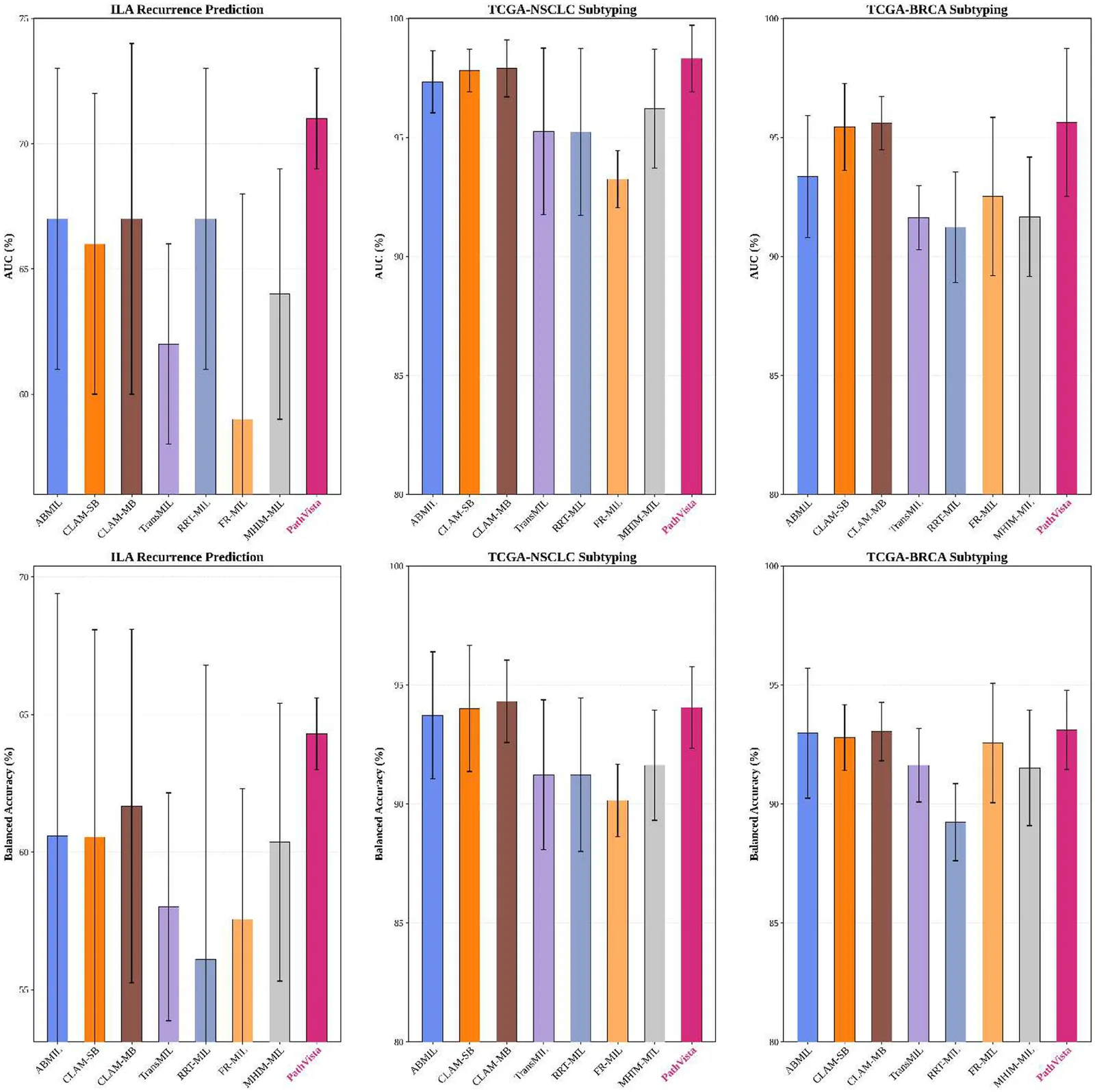
Cross-organ transferability of PathVista for lung recurrence prediction and tumor subtyping. Performance comparison of PathVista with established deep learning methods on non-CRC tasks. **A-C**, AUC comparison across tasks. **D-F**, Balanced accuracy comparison across the same tasks. Error bars indicate standard deviation across five-fold cross-validation.

## Data Availability

The processed datasets and source code generated or used in this study are available from the corresponding author upon reasonable request. Whole-slide images from The Cancer Genome Atlas (TCGA), SURGEN cohort are publicly accessible at https://portal.gdc.cancer.gov/, https://doi.org/10.6019/S-BIAD1285. Gene mutation labels for patients in the TCGA cohort are publicly available from Kather et al. through the DeepHistology repository at https://github.com/jnkather/DeepHistology. The images from CALGB 89803 were obtained directly from the Alliance for Clinical Trials in Oncology, a National Clinical Trials Network cooperative group, and may be requested via Datasharing@alliancenctn.org.

## Code Availability

The underlying code for this study is available in AI4Path PathVista repository and can be accessed via this link: https://github.com/AI4Path-Lab/PathVista.
